# Perceived Discrimination and Psychological Distress: A Survey of Filipinx Americans in Massachusetts during the COVID-19 Pandemic

**DOI:** 10.21203/rs.3.rs-502283/v1

**Published:** 2021-05-07

**Authors:** Dale Dagar Maglalang, Jose L. Condor, Ronna Bañada, Erin Nuestro, Carina Katigbak

**Affiliations:** Stanford University, School of Medicine; Stanford University, School of Medicine; University of Southern California; Boston College; Boston College

**Keywords:** Filipino American, Massachusetts, discrimination, psychological distress, COVID-19

## Abstract

The onset of the COVID-19 pandemic has led to an upsurge of discrimination against Asian American populations, and Filipinx Americans (FA) have reported high cases of perceived discrimination. Prior studies have shown a relationship between experiences of discrimination and poor mental health. The purpose of this study was to examine the association of perceived discrimination and psychological distress among FA living in Massachusetts from a survey collected during the COVID-19 pandemic (N = 133). Multiple regression analysis revealed that experiences of perceived discrimination were associated with psychological distress. Older participants were less likely to report psychological distress. Compared to men, womxn were more likely to report psychological distress. Our findings highlight the potential mental health consequences of perceived discrimination experienced by FA, which may have been exacerbated during the COVID-19 pandemic.

## Introduction

Since the beginning of the COVID-19 pandemic, there has been a significant increase in anti-Asian discrimination in the United States (U.S.). From March 2020 to February 2021, Stop AAPI Hate amassed 3,795 self-reported hate crimes against Asian Americans [1]. Within Asian ethnic groups, Filipinx Americans (FA) were the fourth-leading Asian ethnic group that self-reported experiencing discrimination [1]. One of the horrific attacks on FA occurred in New York City this year - Noel Quintana was riding the subway and was slashed in the face with a box cutter, and received no aid from bystanders on the train [2].

Historically, FA have experienced numerous forms of race-targeted violence. In 1930 in Watsonville, California, a white mob attacked FA men for dancing with white women at a taxi dance hall, which led to the death of one FA [3]. In 1999, Joseph Ileto, a postal worker, was murdered by a white supremacist in Los Angeles, California [4]. These discriminatory experiences can have negative health effects.

Perceived discrimination has been associated with adverse health behaviors and outcomes [5]. This can include poor physical and mental health by increasing the risk for cardiovascular disease, substance use, and symptoms of depression, and psychological distress. Previous studies on FA showed similar associations of perceived discrimination, mental health problems, and substance abuse [6].

There has been limited research on the health outcomes of FA during the pandemic. One of the few studies published found that experiences of perceived discrimination were negatively associated with symptoms of depression and anxiety, though not statistically significant, among FA [7]. This study examined data that was collected during the COVID-19 pandemic, and explored the association of perceived discrimination and psychological distress among FA in Massachusetts.

## Methods

### Data Collection

The data analyzed was from the FA in Massachusetts Health Study. Participants were recruited online through organization listservs and social media platforms. Participants took the survey through Qualtrics and were asked questions about their demographic characteristics, health behaviors and outcomes, and cultural beliefs. Surveys were collected from June through August of 2020. Participants who completed the survey were entered into a raffle to win one of the 15 $15 gift cards. A total of 178 participants took the survey. After excluding incomplete responses, 133 observations were analyzed. This study was approved by the Institutional Review Board of Boston College.

### Measures

#### Psychological Distress

Participants responded to the Kessler Psychological Distress Scale (K10), which measures psychological distress within the last 30 days [8]. The K10 consists of 10 items. Examples include experiences of feeling nervous, hopeless, and depressed. The items are measured on a five-point Likert scale of 1 (None of the time) to 5 (All of the time). The scores are summed with possible ranges of 10–50. The K10’s internal consistency is acceptable (Cronbach’s α = .94).

### Perceived Discrimination

Participants responded to the Everyday Discrimination Scale (EDS), which measures day-to-day experiences of perceived discrimination in the previous 12 months [9]. The EDS consists of 5 items asking about experiences of perceived discrimination. Examples include “receiving poor service” and “treated with less courtesy.” The items are measured on a six-point Likert scale of 1 (Almost everyday) to 6 (Never). The items are reverse coded and averaged with possible ranges of 1–6. The internal consistency for EDS is acceptable (Cronbach’s α = .93).

### Covariates

We included age, gender (man, womxn), sexual orientation (straight, LGBTQ+), education (high school/GED/some college, college/graduate degree), and immigrant status (U.S. born, non-U.S. born) as control variables.

### Analysis

Univariate analysis described the frequency of the distribution of the variables. Following this, Pearson’s correlation and t-tests were conducted to examine correlations and significant differences between the means of the predictors and psychological distress. We then conducted multiple regression to investigate the association of experiences of perceived discrimination and psychological distress controlling for the identified covariates. We checked for the multicollinearity of the model and the mean VIF is 1.18. We handled missing cases using listwise deletion. Data were analyzed using Stata 16 SE.

## Results

The average age of our sample of 133 FA from Massachusetts was 32.15 years old (SD = 12.37). The participants were primarily womxn (71%), straight (77%), holding a college or graduate degree (80%), and U.S. born (54%). (Table 1).

The average mean of psychological distress was 22.67 (SD = 8.71). In examining the predictors, womxn (M = 23.51; SD = 8.56), LGBTQ+ (M = 25.13; SD = 9.18), participants with a high school degree, GED, or some college degree (M = 24.11; SD = 8.41), and U.S. born (M = 23.67; SD = 8.15) had higher mean averages than their counterparts. However, perceived discrimination (*r* = 0.42, *p* < .001) and age (*r*=−0.28, *p* < .001) were the only predictors that were correlated with psychological distress, though the correlations were moderate and weak, respectively.

Participants experienced perceived discrimination on average between less than once a year to a few times a year (M = 2.60; SD = 1.09). In examining the predictors, men (M =2.66; SD = 1.23), LGBTQ+ (M = 2.88; SD = 1.01), participants with a college or graduate degree (M = 2.67; SD = 1.11), and U.S. born (M = 2.67; SD = 1.00) had higher mean averages than their counterparts.

The multiple regression model (Table 2) explained 26.04% of the variance (R^2^ = 0.2940, Adjusted R^2^ = 0.2604, F(6,126) = 8.75, p = 0.000). Experiences of perceived discrimination were positively associated with psychological distress holding all other variables constant (β = 0.41; p < 0.001). Age was negatively associated with psychological distress (β=−0.30; p < 0.001). In addition, compared to men, womxn are positively associated with psychological distress (β = 0.41; p < 0.05).

## Discussion

Our study found that discrimination is associated with psychological distress among our sample even after controlling for confounders. This finding is generally consistent with prior studies that examined similar constructs among FA [6]. However, our results differ from Woo and Jun’s findings [7] that found a negative association of perceived discrimination and depressive symptoms among FA during the COVID-19 pandemic. This suggests that overall experiences of perceived discrimination may have been further compounded by the racialization of the COVID-19 virus, thereby contributing to poorer mental health among FA.

We also found that age is negatively associated with psychological distress. A potential explanation for this finding is that older participants may have support networks and coping strategies that buffer or alleviate experiences of psychological distress.

FA womxn are also more likely to report psychological distress compared to men. The fetishization of FA womxn to be subservient and docile through the traditional Maria Clara image [10] may contribute to experiences of psychological distress among womxn. Interestingly, FA men reported a slightly higher mean of perceived discrimination but FA womxn reported a higher mean of psychological distress. This may suggest that there are gendered differences in how FA womxn experience and internalize stress compared to FA men.

### New Contributions to the Literature

To date, there is little to no data on the experiences of perceived discrimination and mental health among FA in Massachusetts. Furthermore, the time period in which the study was conducted may help provide insight on the mental health effects of perceived discrimination that occurred before and during the COVID-19 pandemic among FA. This can inform mental health providers, researchers, and politicians to implement interventions and policies that addresses discrimination experienced by racial and ethnic minorities in this geographic location and during a pandemic.

### Limitations

A limitation to this study is that the measure of perceived discrimination includes the last 12 months from which the participants took the survey, indicating that their experiences of perceived discrimination may have occurred before or during the COVID-19 pandemic. However, the K10 scale asked about experiences of psychological distress within the last 30 days from when the participants took the survey. Nevertheless, the continuing rise of anti-Asian discrimination highlights the importance of this research. Another limitation is that the participants were from Massachusetts, thus, generalizations cannot be made to all FA in the U.S.

## Figures and Tables

**Table 1:** Psychological distress, perceived discrimination, and socio-demographic characteristics of Filipinx Americans in Massachusetts (N = 133)

	Percentage or Mean (Standard Deviation)	Mean (Standard Deviation) of Psychological Distress	Mean (Standard Deviation) of Discrimination	p-value^a^
Psychological Distress	22.67 (8.71)	-	-	
Discrimination	2.60 (1.09)	-	-	<0.001***
Age (18–71 years old)	32.15 (12.37)	-	-	<0.001***
Gender				0.084
Men	29.32	20.64 (8.09)	2.66 (1.23)	
Womxn	70.68	23.51 (8.56)	2.57 (1.03)	
Sexual Orientation				0.078
Straight	77.44	21.95 (8.48)	2.51 (1.10)	
LGBTQ+	22.56	25.13 (9.18)	2.88 (1.01)	
Education				0.337
High School/GED/Some College	20.30	24.11 (8.41)	2.31 (.95)	
College/Graduate Degree	79.70	22.30 (8.78)	2.67 (1.11)	
Immigrant Status				0.152
U.S. Born	54.14	23.67 (8.15)	2.67 (1.00)	
Non-U.S. Born	45.86	21.49 (9.25)	2.51 (1.18)	

* p < 0.05

** p<0.01

*** p<.001

a. Pearson’s correlation or t-test p-values

**Table 2:** Association of experiences of perceived discrimination and psychological distress among Filipinx Americans in Massachusetts (N = 133)

	B	SE	β
Discrimination	3.27	0.61	0.41***
Age (18–71 years old)	−0.21	0.06	−0.30***
Gender (ref. Men)			
Womxn	3.70	1.46	0.19*
Sexual Orientation (ref. Straight)			
LGBTQ+	2.23	1.59	0.11
Education (ref. High School/GED/Some College)			
College/Graduate Degree	−0.45	1.85	−0.02
Immigrant Status (ref. U.S. Born)			
Non-U.S. Born	0.95	1.43	0.05

* p < 0.05

** p<0.01

*** p<.001

## References

[1] Jeung R, Yellow Horse A, Popovic T, Lim R. Stop AAPI Hate National Report 3/19/20 – 2/28/21 [Internet]. 2021. Available from: https://secureservercdn.net/104.238.69.231/a1w.90d.myftpupload.com/wp-content/uploads/2021/03/210312-Stop-AAPI-Hate-National-Report-.pdf

[2] Mendiola R. Filipino American Man Slashed In the Face While Riding NYC Subway [Internet]. Asian J. 2021. Available from: https://www.asianjournal.com/usa/newyork-newjersey/filipino-american-man-slashed-in-the-face-while-riding-nyc-subway/

[3] De Witt HA. The Watsonville Anti-Filipino Riot of 1930: A Case Study of the Great Depression and Ethnic Conflict in California. South Calif Q [Internet]. [University of California Press, Historical Society of Southern California]; 1979;61:291–302. Available from: http://www.jstor.org/stable/41170831

[4] Sanchez R. L.A. Shooting Suspect Faces State, U.S. Charges [Internet]. Washington Post. 1999. Available from: https://www.washingtonpost.com/wp-srv/national/longterm/hatecrimes/stories/furrow081399.htm

[5] Williams DR, Lawrence JA, Davis BA, Vu C. Understanding how discrimination can affect health. Health Serv Res. United States: Health Research and Educational Trust; 2019;54:1374–88. 31663121 10.1111/1475-6773.13222 PMC6864381

[6] Tsai JH, Thompson EA. Impact of social discrimination, job concerns, and social support on filipino immigrant worker mental health and substance use. Am J Ind Med. Hoboken, NJ: Wiley-Liss; 2013;56:1082–94. 23794397 10.1002/ajim.22223

[7] Woo B, Jun J. COVID-19 Racial Discrimination and Depressive Symptoms among Asians Americans: Does Communication about the Incident Matter? J Immigr Minor Heal [Internet]. 2021; Available from: 10.1007/s10903-021-01167-x PMC7936603 33677775

[8] Kessler RC, AndrewsS G, Colpe LJ, Hiripi E, Mrroczek DK, Normand S-LT, Short screening scales to monitor population prevalences and trends in non-specific psychological distress. Psychol Med [Internet]. 2002/09/26. Cambridge University Press; 2002;32:959–76. Available from: https://www.cambridge.org/core/article/short-screening-scales-to-monitor-population-prevalences-and-trends-in-nonspecific-psychological-distress/F141675CCD0E08C0FB98E01C006B4E0D 10.1017/s0033291702006074 12214795

[9] Sternthal MJ, Slopen N, Williams DR. Racial Disparities in Health: How Much Does Stress Really Matter? Du Bois Rev Soc Sci Res Race [Internet]. 2011/04/15. Cambridge University Press; 2011;8:95–113. Available from: https://www.cambridge.org/core/article/racial-disparities-in-health/43F0CAE561A71DAC646E9E8014172D1A 10.1017/S1742058X11000087 PMC5993442 29887911

[10] Cruz D. Transpacific Femininities: The Making of the Modern Filipina. Durham: Duke University Press; 2012.

